# Nested association mapping reveals the genetic architecture of spike emergence and anthesis timing in intermediate wheatgrass

**DOI:** 10.1093/g3journal/jkab025

**Published:** 2021-01-30

**Authors:** Kayla R Altendorf, Steven R Larson, Lee R DeHaan, Jared Crain, Jeff Neyhart, Kevin M Dorn, James A Anderson

**Affiliations:** 1 USDA-ARS, Forage Seed and Cereal Research Unit, Irrigated Agriculture Research and Extension Center, Prosser, WA 99350, USA; 2 The Land Institute, Salina, KS 67401, USA; 3 USDA-ARS, Forage Range and Research Lab, Utah State University, Logan, UT 84322, USA; 4 Department of Plant Pathology, Kansas State University, Manhattan, KS 66506, USA; 5 GEMS Informatics Initiative, University of Minnesota, St. Paul, MN 55108, USA; 6 USDA-ARS, Soil Management and Sugarbeet Research, Fort Collins, CO 80526, USA; 7 Department of Agronomy and Plant Genetics, University of Minnesota, St. Paul, MN 55108, USA

**Keywords:** Nested association mapping, intermediate wheatgrass, flowering time

## Abstract

Intermediate wheatgrass (*Thinopyrum intermedium*) is an outcrossing, cool season grass species currently undergoing direct domestication as a perennial grain crop. Though many traits are selection targets, understanding the genetic architecture of those important for local adaptation may accelerate the domestication process. Nested association mapping (NAM) has proven useful in dissecting the genetic control of agronomic traits many crop species, but its utility in primarily outcrossing, perennial species has yet to be demonstrated. Here, we introduce an intermediate wheatgrass NAM population developed by crossing ten phenotypically divergent donor parents to an adapted common parent in a reciprocal manner, yielding 1,168 F_1_ progeny from 10 families. Using genotyping by sequencing, we identified 8,003 SNP markers and developed a population-specific consensus genetic map with 3,144 markers across 21 linkage groups. Using both genomewide association mapping and linkage mapping combined across and within families, we characterized the genetic control of flowering time. In the analysis of two measures of maturity across four separate environments, we detected as many as 75 significant QTL, many of which correspond to the same regions in both analysis methods across 11 chromosomes. The results demonstrate a complex genetic control that is variable across years, locations, traits, and within families. The methods were effective at detecting previously identified QTL, as well as new QTL that align closely to the well-characterized flowering time orthologs from barley, including *Ppd*-*H1* and *Constans*. Our results demonstrate the utility of the NAM population for understanding the genetic control of flowering time and its potential for application to other traits of interest.

## Introduction

Intermediate wheatgrass (*Thinopyrum intermedium;* (Host) Barkworth& D.R. Dewey; IWG hereafter) is a perennial, cool-season grass undergoing direct domestication as a dual-use forage and grain crop for human consumption (DeHaan *et al.* 2014). IWG is primarily self-incompatible, outcrossing allohexaploid (2n = 6x = 42) (Dewey 1962). Native to Europe and Asia, it was introduced to North America in 1932 and has since been used primarily as a hay and pasture grass (Dewey 1962; Ogle *et al.* 2011) and a source disease resistance genes for common wheat (*e.g.*, Brettell *et al.* 1988; Friebe *et al.* 1996; Turner *et al.* 2013). Citing the need for perennial crops to improve agricultural sustainability on highly erodible or marginal lands, the Rodale Institute (Kutztown, PA, USA) in the early 1980s surveyed over 100 perennial grasses for their domestication potential (Wagoner 1990). They selected IWG due to its relatively large seed size, nutritional similarity to wheat, perennial growth, and its ability to be mechanically harvested. Initial germplasm surveys began in 1987 (Wagoner 1990) after which, breeding and domestication programs were established at The Land Institute (TLI; Salina, KS; DeHaan *et al.* 2018), the University of Minnesota (Zhang *et al.* 2016), the University of Manitoba (DeHaan *et al.* 2014), and new programs have been initiated in Utah and Internationally in Europe in the last few years. These programs utilize phenotype-, pedigree-, or genotype-based recurrent selection and cultivars are developed as synthetics (DeHaan *et al.* 2014; Zhang *et al.* 2016; Bajgain *et al.* 2020a, b; Crain *et al.* 2020).

Despite the many genetic resources developed for IWG, and some early selection success, breeding targets remain vast. IWG has a dense genetic consensus map with 10,029 markers from seven full-sib families (Kantarski *et al.* 2016), an optimized protocol for developing genotyping-by-sequencing (GBS) libraries (Elshire *et al.* 2011; Zhang *et al.* 2016), and a draft reference genome under development by the *T. intermedium* Genome Sequencing Consortium (DeHaan *et al.* 2018; Larson *et al.* 2019). Breeding and selection have primarily focused on increasing seed size and yield on a per spike basis (DeHaan *et al.* 2018) and despite the infancy of breeding efforts, gains from selection have been observed. For example, after 5 cycles of selection at TLI, a 143% increase in yield per spike and a 60% increase in seed mass was predicted (observed response would require evaluation in the same environment) in a spaced plant setting (DeHaan *et al.* 2018). Furthermore, genomic selection has shown great promise in IWG, demonstrating high-predictive ability (in one study, *r*= 0.46 – 0.67; depending on the trait, and > 0.5 in another) and has improved the precision and efficiency of selection (Zhang *et al.* 2016; Crain *et al.* 2021). Including significant markers from QTL mapping studies as co-factors in genomic selection models has been shown to increase predictive ability and increases the frequency of favorable alleles (Zhang *et al.* 2017; Bajgain *et al.* 2019a, b). However, IWG is still susceptible to seed shattering (Larson *et al.* 2019), has low threshability (Zhang *et al.* 2016), is tall and prone to stem lodging (Frahm *et al.* 2018), and has low-floret site utilization that may contribute to low yields (Altendorf *et al.* 2020). An improved understanding of the genetic architecture of these important traits has the potential to further increase efficiency and precision in IWG breeding efforts through genomics assisted breeding, but the difficulty lies in the fact that relatively few marker-trait association studies have been conducted in IWG and the genetic control of many important traits remains poorly understood.

Nested association mapping (NAM) was developed to combine the benefits of both linkage and association mapping by crossing one common parent with a series of diverse donor parents and developing segregating populations in the form of recombinant inbred lines (RILs; Yu *et al.* 2008). This approach has demonstrated utility in many crop species including maize (Buckler *et al.* 2009; McMullen *et al.* 2009), rice (Fragoso *et al.* 2017), wheat (Bajgain *et al.* 2016; Jordan *et al.* 2018; Wang *et al.* 2019), barley (Maurer *et al.* 2016; Nice *et al.* 2017; Hemshrot *et al.* 2019), sorghum (Bouchet *et al.* 2017), and soybean (Song *et al.* 2017). Notably, all the aforementioned species are self-compatible where the development of RILs is possible and commonplace. To our knowledge, there have been no formally published NAMs developed in an outcrossing, self-incompatible species, nor for any cool season, perennial grasses (Talukder and Saha 2017).

In an outcrossing, self-incompatible species, the NAM design still includes common and donor parents, but eliminates the RIL development step, and all progeny are F_1_ and therefore highly heterozygous and heterogeneous. While this may result in a decrease in mapping resolution due to the lack of recombination and breakdown of LD associated with inbreeding, there are various practical advantages NAM that may be realized. First and foremost, multiple parents offer more genetic variation and increase the utility of a population by allowing the dissection of more than a single trait compared with a traditional bi-parental mapping population. Second, relative to genome wide association study (GWAS) in diverse populations, NAM offers increased frequency and sampling of rare alleles in both common and donor parents that may otherwise go undetected or filtered out due to a minimum minor allele frequency (MAF) threshold. Finally, crossing wild or unadapted donor parents with a highly adapted common parent allows for the assessment of diverse germplasm (Poland *et al.* 2011; Nice *et al.* 2017).

Contrary to an inbred NAM, an F_1_ NAM requires special considerations with regards to the segregation of alleles. For example, bi-parental families can segregate from anywhere between 2 and 4 alleles, and this number can vary across loci (Van Ooijen 2011). Thus, it is not possible to observe allele combinations in homozygous, identical-by-descent states as it is in an inbreeding species. Because the parents are segregating, imputation of densely genotyped parents on progeny is difficult, and diverse donor alleles cannot be assessed relative to a common genetic background. Many computational programs developed for NAMs and other multi-parent populations, including the R packages “NAM” (Xavier *et al.* 2015), “mppR” (Garin *et al.* 2107), and “R/qtl2” (Broman *et al.* 2019), all depend on homozygous parents and progeny to reconstruct haplotypes and phasing and are not suited for heterozygous parents. In some cases, heterozygous sites within the parents and progeny are excluded from the analysis, which would likely result in significant data loss in the case of an outcrossing species. Finally, it is important to note that instead of maximizing genetic divergence between parental material, as would be the case in a traditional NAM, the objective instead is to maximize heterozygosity within parents to observe segregation among the progeny. These challenges can be overcome by using programs and methods designed for phasing and mapping in outcrossing species, such as JoinMap and MapQTL (Van Ooijen 2009; 2011). Considering the demonstrated utility of the population design and the need within the IWG breeding community to assess the genetic control of many traits of interest, a NAM population is worth exploring.

Here, we introduce an IWG NAM population and assess its utility by dissecting the genetic control of flowering time. The timing of the transition from vegetative to reproductive phases is a critical step to ensure proper timing for pollination, seed set and dispersal, and variation for this trait has played a critical role in the adaptation of crops to new growing environments (Cockram *et al.* 2007). The objectives of this work were to: (1) develop and genetically characterize an IWG NAM population; (2) describe the phenotypic variation in the NAM for flowering time over 2 years in two distinct locations: St. Paul, MN, and Salina, KS; (3) assess the genetic control of flowering time using two approaches: GWAS and linkage mapping within and across multiple populations. The linkage mapping method accurately assesses the within family allele effects by utilizing phasing and tracing the parental origin of 2–4 alleles at a locus. The GWAS approach serves as additional support for QTL identification, and results are compared across both methods.

## Materials and methods

### Population development and establishment

Ten phenotypically diverse genets (donor parents) and one low-shattering genet (common parent) were identified from Cycle 2 of the University of Minnesota breeding program based on their traits of interest (Table 1). Genet refers to plants of the same genetic makeup, with multiple clones of each genet are referred to as ramets. This terminology is consistent with Zhang *et al.* (2016) and accounts for the heterozygous nature of IWG as the terms line or cultivar would be inconsistent with normal usage in inbred crops. Parents, and therefore families, were named after their numerical designation within the IWG breeding program preceded with “WGN” for “Wheatgrass NAM.” Parental genets were propagated from the field in Fall 2015 into 3–5 clones each, planted in 3.8 L pots, and allowed to re-establish in the greenhouse before vernalization at 4°C for 2 months. After vernalization, plants were placed in a growth chamber (16 hour day, 18–20°C) to induce flowering. In May 2016, multiple reciprocal crosses were made between each donor parent and the common parent by bagging spikes together in custom pollination bags (PBS International, UK) just before pollen shed. Spikes from each cross were harvested in July 2016 and kept separate on the basis of family and maternal parent identity. Spikes were threshed and cleaned using a belt thresher, sieve (12/64” round; SEEDBURO, Des Plaines, IL, USA) and aspirator (Air Blast Seed Cleaner; ALMACO, Nevada, IA, USA). Approximately 150 seeds from each cross (∼75 from each maternal parent) were placed on moist blotter paper (Anchor Paper, St. Paul, MN, USA) in petri dishes and subjected to a cold treatment of 4°C for 3–5 days or until germination occurred. Germinating seeds were transplanted at 1 cm depth into 10 cm 21-count trays and placed in a misting greenhouse for 4 days and then transferred to an outdoor nursery. Plants were watered daily and fertilized weekly with a standard solution (15 mL per 4.4 L) of 20-20-20 fertilizer, and monthly with slow-release Osmocote (The Scotts Company, Marysville, OH, USA). In August, plants were clipped to ∼5 cm to promote tillering, propagated into four ramets per genet and placed into 5 × 5 cm peat pots (Plantation Products, Norton, MA, USA) in September. Genets were completely randomized within blocks and were transplanted into spaced plant nurseries in a randomize complete block design (RCBD) with two blocks at the University of Minnesota Agricultural Experiment Station in St. Paul, MN, USA (1-m centers; STP hereafter) using a mechanical transplanter on 30 September, and at The Land Institute in Salina, KS (0.9-m centers; TLI hereafter) using a jab-type planter on 16 October. The genets, or spaced plants, were considered the experimental units. Several clones of each donor parent (∼3 per block) and the common parent (∼25 per block) were included and a two-plant border was established to limit edge effects. Transplants were watered once after transplanting to promote successful establishment. Plots were hand weeded and cultivated with a multivator (Ford Distributing, Marysville, OH, USA) as necessary to control weeds. In addition, a pre-emergent herbicide, Dual II Magnum (S-metolachlor, Syngenta US), was applied at STP in April 2017 and May 2018 at a rate of 1.75 L ha^−1^. Herbicides were not used at TLI. At both locations, plots were mowed to 15 cm height after harvest and fertilized with urea (56.0 kg ha^−1^ at STP; 78.5 kg ha^−1^ at TLI) in fall 2017. Forty-four ramets died between transplanting and the end of the 2017 harvest season at TLI, and 25 additional ramets died in the 2018 field season. An additional 203 ramets at TLI were deemed not worth harvesting in 2018 due to drought conditions. In St. Paul, 76 ramets were lost between transplanting and harvesting in 2017, and an additional 2 in 2018.

**Table 1:** Families of the intermediate wheatgrass nested association mapping population, their family sizes separated by maternal parent, and their phenotypic characteristics as recorded in historical breeding program data from St. Paul, MN, which served as the basis of their initial selection

		Family size^a^ and maternal parent			Phenotypic characterization				
Parent	Parent type	Common	Donor	Total	Heading date (1–5)^b^	Height (cm)	Seed size (mg)	Shattering (0–4)^c^	Threshability (1–9)^d^
WGN07	Donor	59	62	121	3	75	6.83	1	7
WGN15	Donor	60	60	120	5	130	8.88	0	6
WGN26	Donor	62	59	121	3	135	12.52	3	1
WGN36	Donor	51	63	114	3	103	10.1	0.5	4
WGN38	Donor	66	29	95	2	77	9.1	1	0.5
WGN39	Donor	59	63	122	3	131	9.5	3	3
WGN45	Donor	54	64	118	—	120	10.58	0.5	5
WGN46	Donor	58	63	121	—	108	8.28	3	6
WGN55	Donor	61	62	123	—	111	10.48	3	6
WGN59	Common	—	—	—	—	131	10.56	0.5	7
WGN63	Donor	57	56	113	—	126	9.18	3	1

a Determined by samples for which there is both phenotypic and genotypic data.

b Historical data on the heading date was not available for all parents, where one is late and five is early heading.

c Shattering scale, where zero is low and four is high.

d Threshability scale, where zero is low and nine is high.

### Growing degree days

Weather data for St. Paul was obtained from National Oceanic and Atmospheric Administration (NOAA, RRID: SCR_011426) from the St. Paul Agricultural Experiment Station (Station ID: USC00218450). Weather data for Salina, KS was obtained from the TLI Weather Station. Growing degree days (GDDs) were calculated in degrees Celsius using the following equation, where *T*_max_ and *T*_min_ are the maximum and minimum daily temperatures, and *T*_base_ is 0°C, or the base temperature for growth used in IWG (Frank 1996; Jungers *et al.* 2018):

$$\left[\left(T_{\text{max}}+T_{\text{min}}\right)\;/2\right]-T_{\text{base}}$$

A maximum threshold of 37°C was set for *T*_max,_ which is the predicted maximum temperature for growth in wheat (Porter and Gawith 1999), and GDD accumulation began and ended after 5 consecutive days where the average daily temperature exceeded *T*_base_ (Frank and Hoffman 1989).

### Phenotypic data collection

Two measures of reproductive growth stage were recorded: spike emergence percent and anthesis stage. IWG spike length is variable both within and among genets, making it challenging to visually estimate the proportion of the spike that has emerged from the boot as is done in a traditional maturity rating scale such as BBCH, Feekes, or Zadoks (Large 1954; Zadoks *et al.* 1974; Lancashire *et al*. 1991). Thus, to more precisely capture variation in emergence time, when spikes were approximately 50% emerged on average across the population, the length of the spike emerged from the boot (from the tip of the most apical spikelet to the base of the flag leaf) was measured in cm on one spike per plant. As maturity within a plant can vary, especially in the first year, larger, more uniform spikes were chosen for measurement to minimize experimental error (DeHaan *et al.* 2018). In cases of high within-plant variability (*e.g.*, ∼5 cm), the mean of two spikes was recorded. Harvesting procedures were previously outlined in Altendorf *et al.* (2020). After harvest, three spikes were aligned end-to-end (from tip of most apical spikelet to base of the most basal spikelet, a spikelet defined as having glumes and at least one floret) along a measuring tape and total length was recorded and divided by three to obtain the mean. Length emerged divided by final spike length represents percent spike emergence. In cases where the spike was completely emerged, the length of the emerged peduncle was included, resulting in some estimates exceeding 100%. Early spike emergence is not always coupled with early anthesis in IWG (personal observation). Thus, Feekes flowering time was also recorded for each plant on one occasion per environment when anthers were showing (approximately 1600–1800  hour) and when approximately 50% of the plants were in anthesis (Large 1954). Categories were coded as ordinal variables for data analysis and included: (1) boot stage where no spike is visible; (2) heads emerging or < 25%; (3) heading 25%; (4) heading 50%; (5) heading 75%; (6) heading complete but no anthers visible; (7) beginning flowering, where yellow anthers are beginning to emerge at the center of the spike; (8) flowering 50%, where anthers are visible through the center and top of spike; (9) flowering 100% where anthers (possibly white or dehiscing) are visible throughout the entirety of the spike including the most basal spikelets; and (10) kernels watery ripe, where anthers are likely dehisced and florets appear plump. The same person recorded anthesis across all locations and years. Dates of data collection events are reported in Supplementary Figure S1.

### Phenotypic data analysis

All phenotypic data analyses were conducted in R v3.6.1 (R Core Team 2020). In a separate analysis of yield component traits, we previously reported the calculation of estimated marginal means (“emmeans”) for each genet nested within family for both spike emergence percent and anthesis, as well as correlations between traits, and both broad and narrow sense heritability estimates (Lenth 2018; Altendorf *et al.* 2020). Because an initial linear model analysis across environments revealed highly significant interactions, we analyzed all unique year by location combinations (*n *=* * 4; environments hereafter) separately. This decision was further supported by differences in plant age between 2017 and 2018, as well as major differences in climate and GDD accumulation between STP and TLI, and a severe drought in 2018 at TLI (Altendorf *et al.* 2020). Parental means were calculated and plotted alongside emmeans for progeny genotypes using ggplot2 (Wickham 2016). To test whether divergent parental phenotypes produced variable progeny, a correlation analysis was conducted between the differences in parental phenotypes and the standard deviation among the progeny on an individual environment basis. To test for a maternal effect, a *t*-test was conducted between progeny derived from the common and donor parents as the mother within a cross. The relationship between the two forms of flowering time data were tested using linear and quadratic model fits.

### Genotyping-by-sequencing

Young leaf tissue was harvested from each genet prior to planting, freeze dried, and genomic DNA was extracted using the BioSprint 96 Plant DNA Kit (QIAGEN, the Netherlands). DNA was quantified using QuantiFluor dsDNA System (Promega Corporation, WI, USA) and normalized to 10 ng/µl. Genotyping by sequencing libraries were developed using *PstI*/*Msp* I enzymes following Zhang *et al.* (2016) with two barcodes per sample. Every 96 samples were pooled, creating a total of fifteen 96-plex libraries. The common parent was sampled eight times and the donor parents six each to achieve higher sequencing depth. Libraries were amplified and cleaned using the QIAquick PCR Purification Kit (QIAGEN, the Netherlands), quality control was done using Picogreen (ThermoFisher Scientific, MA, USA), Agilent Bioanalyzer (Agilent, CA, USA) and Kapa qPCR, and subjected to size selection of 160–240bp using PippinHT (3% agarose). Each pool was sequenced in a single lane of a 100 bp single read run on the Illumina HiSeq 2500 HO using v4 chemistry at the University of Minnesota Genomics Center.

### Variant detection and filtering

Fastq files from the sequencer were demultiplexed using the Barcode Splitter tool from the FastX-Toolkit (RRID: SCR_005534) where barcodes were matched at the beginning of reads and no mismatches were allowed. Read quality was assessed using FastQC (RRID: SCR_014583). Adapter sequences, designed according to (Poland *et al*. 2012), were removed using CutAdapt (Martin, 2011; RRID: SCR_011841). The Quality Trimmer tool from FastX-Toolkit was used to trim reads with a quality score less than 30 (phred + 33 scale) and a minimum read length of 30 (-Q 33 -t 30 -l 50 -v). After adapter removal and read trimming, quality was confirmed by re-running FastQC. Bowtie2 (Langmead and Salzberg, 2012; RRID: SCR_055476) was used to align reads to v2 of the draft IWG reference genome (access provided by The *T. intermedium* Genome Sequencing Consortium), which was indexed prior to analysis. Options were set to require reads to align entirely and the very-sensitive preset was used, with zero ambiguous reference characters. Reads were filtered to include only those that mapped uniquely to the reference, and files were sorted and indexed using SAMtools (Li *et al.* 2009; RRID: SCR_002105). Within each lane, fastq files with barcodes corresponding to the same sample were concatenated using a custom BASH script. The Genome Analysis Toolkit (GATK) v4.1.2 (McKenna *et al.* 2010; RRID: SCR_001876) was used to call variants, beginning with the HaplotypeCaller tool, which was set to eliminate the duplicate read filter, run in GVCF mode and with an expected heterozygosity rate of 0.01. GATK required a dictionary and an “fai” index of the reference genome; these were created using the GATK “CreateSequenceDictionary” and the SAMtools “faidx” commands, respectively. The CombineGVCF tool was used to merge individual sample gvcf files in a hierarchical manner. GVCF files from 18 samples were small in size (∼4% of the average), indicating low coverage, and because they caused significant computational delays in the CombineGVCF stage they were removed. The GenotypeGVCF tool was used to perform joint genotyping. The program GNU Parallel (Tange 2011) facilitated parallel calculations throughout the pipeline.

SNP filtering was done using VCFTools v0.1.16 (Danecek *et al.* 2011; RRID: SCR_001235) to include only bi-allelic SNPs with a maximum of 20% missing data, a minimum allele depth of 5, and a MAF greater than 0.005. A low MAF filter was used to allow rare alleles to segregate in any single family at a rate of 0.05, divided by 10 families. Missingness on an individual basis was calculated, and those that exceeded 70% were removed from the population. The program Cervus v3.0 (Kalinowski *et al.* 2007) was used to identify unintended outcrosses and progeny derived from self-pollination using 2,500 markers (larger number of markers can exceed program limitations) with the lowest percent missing data. Fathers were predicted using the known mothers, then the analysis was rerun using predicted fathers as known and predicting mothers. Progeny derived from self-pollination or outcrosses (those with unexpected parents resulting from stray pollen or seed contamination) were removed from the population.

### Marker imputation, population structure and linkage disequilibrium

LinkImpute was used for marker imputation on progeny only (Money *et al.* 2015). The LD-kNNi method chooses k-nearest neighbors based on LD between SNPs and is specifically designed to handle data from highly heterozygous species (Money *et al.* 2015). Imputation accuracy was tested by masking and imputing 10,000 random known genotypes. The program STRUCTURE v 2.3.4 (Pritchard *et al.* 2000) was used to assess population structure using K = 1 through 10, with five replicates each with a length of 25,000 for a burn-in period, followed by 75,000 MCM reps. Optimum *K* values were assessed using Structure Harvester (Earl and vonHoldt 2012) based on the maximum Delta *K* value using the Evanno method. The selected K CLUMPP “indfile” was imported into CLUMPP v1.1.2 (https://rosenberglab.stanford.edu/clumpp.html) to develop an optimal Q matrix over the 5 replicates. In general, LD is expected to decay as the genetic or physical distance increases and as the number of generations or cycles of recombination increases. Pairwise LD was estimated using the squared correlation coefficient *r*^2^ for pairs of markers within a chromosome and family using the makeGenotypes and LD commands in the “genetics” package (Warnes *et al.* 2019). Pooled *r*^2^ values across all families were plotted over cM distance and the relationships were modeled using a spline approach (Vos *et al.* 2017) in the “segmented” package (Muggeo 2008). The extent of LD was estimated when the fitted line intersected with *r*^2^ = 0.2 (Zegeye *et al.* 2014). Principal components analysis (PCA) was conducted using the “rrblup” package a.mat function (Endelman 2011).

### Genetic map creation

We created a consensus map for each of the 21 chromosomes of the NAM using JoinMap v5 (Van Ooijen 2011; RRID: SCR_009248). Because parents were genotyped 6–8 times each, the mode call across all samples was selected for the parental genotype at each locus using a custom R script and the “vcfR” package (Knaus and Grünwald 2017). Families were separated, a MAF filter of minimum 0.05 was applied, and loci with greater than 20% missing data were removed. Markers were filtered within each family to include only those that segregated as two heterozygous parents (hkxhk), or as heterozygous in the common parent only (lmxll) or heterozygous in the donor parent only (nnxnp) (Van Ooijen 2009). In highly heterozygous species, genotyping by sequencing data is susceptible to false homozygous calls because both alleles are required to randomly anneal with an adapter, amplify, sequence, align, and pass quality filters to correctly call the locus. JoinMap does not tolerate these low frequency sequencing or calls errors (*e.g.*, an nnxnp locus with a progeny genotype pp). We calculated the genotype frequency at each locus; if an “impossible” genotype (pp in this example) persisted at a frequency of less than 0.05, it was assumed to be an erroneous homozygous call and was changed to a heterozygote. If the erroneous calls persisted at a rate of greater than 0.05 at a specific locus, the locus was removed. JoinMap .loc files were created on a per family and per chromosome basis, allowing chromosomes from the physical sequence map to serve as anchors for grouping markers. Within each family, locus genotype frequency was calculated and any markers that displayed a significant level (α =  0.1) of segregation distortion were excluded. The groupings tree was calculated and a single group with the highest number of markers was selected at a minimum LOD of 4. Each family group was selected, and consensus maps were calculated using Combine Groups for Map Integration with the Regression Mapping option which conducts three attempts, or rounds, of map creation. More than 250 markers per map proved to be computationally intensive, and thus when more were present, Calculation Options were set to exclude the third round of mapping. If insufficient linkages were detected, the LOD threshold in calculation options was lowered by increments of 0.1. To extract phased marker data for each family, all previously excluded markers were unselected, genotype frequency was recalculated and only markers with highly significant (α =  1.0 × 10^−6^) distortion were excluded. Groups were created using the map node from Round 3 (or Round 2 in cases with 250+ markers) maps, the Maximum Linkages tab was calculated to phase the loci, allowing markers that were initially excluded to phase if they were present on the consensus map. The quality and order of the chromosome map was assessed by correlating cM positions of markers shared with another IWG consensus map (Kantarski *et al.* 2016). Several linkage groups were created in JoinMap in reverse order and to maintain consistency across other studies in IWG, maps from these LGs were inverted. The final result of this process was one .map file comprised of a list of marker names and marker positions in 21 linkage groups, herein referred to as the NAM Consensus Map (or NAM Consensus .map file).

### Genome wide associations

Genomewide association mapping was conducted using GAPIT software (Lipka *et al.* 2012) with the default kinship matrix calculation and a MAF of 0.005. STRUCTURE results indicated optimal *K* = 8 (Supplementary Figure S2). Results in GAPIT with and without the Q matrix had very similar results (*r *=* * 0.965) and therefore Q was not used. No additional PCs were added as the model selection feature within GAPIT showed PC = 0 to have the highest BIC for all traits and environments. Markers with *p*-value threshold of 0.00025 (LOD = 3.6) were considered significant. QTL within clusters or peaks were resolved using the mmer function in the R package “Sommer” (Covarrubias-Pazaran 2016). A multi-locus model was fitted by including significant markers from the genomewide scan as fixed effects and genets as random effects; the covariance between genets was modeled using the realized additive kinship matrix. Markers clustered on a chromosome within significant QTL peaks were iteratively removed from the model if they were insignificant (*p* > 0.001) or within a 21 cM window of a more significant or frequently detected marker. Variance explained by significant QTL was calculated from the model output. Allele frequencies and effects were obtained from the GAPIT output.

### QTL linkage mapping

Using a custom R script, Round 2 (as recommended in the JoinMap v5 program manual) consensus maps for each chromosome and locus files, described above, were utilized in MapQTL v6 (Van Ooijen 2009; RRID: SCR_009284). A full analysis of the cross-pollinated (CP) populations yielded excessive singularity errors, which can occur when there are regions of the map containing markers only from one parent [i.e., common parent lmxll markers, or donor parent nnxnp markers (Van Ooijen 2009)]. In our analysis, this was likely due to long stretches of markers from the common parent. Therefore, the two-way pseudo testcross model (TWPT) was used to analyze QTLs within families and combined over all families for each location and year (environment, *n *=* * 4) separately. The TWPT approach simplified the analysis by splitting each linkage group into a common parent linkage group and a donor parent linkage group and using a single-parent Doubled Haploid model for QTL analysis (Van Ooijen 2009). In this case, 21 linkage groups from the NAM Consensus .map file (.map list of markers and positions) were divided into a second map, herein referred to as the TWPT Map (or TWPT .map file) containing 21 linkage groups for the common parent (lmxll markers only) and 21 linkage groups for the donor parent (nnxnp markers only). The hkxhk markers were removed and heterozygous genotypes (lm & np) were converted to A and homozygous genotypes (ll & nn) to B. The maximum likelihood mixture analysis procedure was used with maximum 20 iterations, with a maximum number of neighboring markers of 10. For each analysis, a permutation test with 1,000 iterations was conducted to determine within-family and combined genome-wide significance thresholds. Each LOD threshold for each family or combined analysis within environment was applied accordingly. Interval mapping was conducted first, and significant LOD peaks, a maximum of one per LG, were selected as cofactors. The Automatic Cofactor Selection procedure was used to determine the final set of cofactors which were used in a single round of restricted multiple QTL mapping (rMQM). A two-LOD drop off interval was used to establish QTL intervals (Van Ooijen 2009) and was determined using a custom R script.

### Visualization of results and proximity to orthologous genes

Marker position, both physical from the reference genome and genetic from the NAM consensus map across all linkage groups, were normalized and visualized using “LinkageMapView” (Ouellette *et al.* 2018). Markers in common between both the physical and the NAM genetic map were connected with a line using the posonleft function. As IWG linkage groups have shown high collinearity with barley (Kantarski *et al.* 2016; Zhang *et al.* 2016) known flowering time orthologs were selected (Supplementary Table S1) and aligned to the IWG draft reference genome using BLASTN 2.6.0+ (Altschul *et al.* 1997) with the online BLAST resource (https://phytozome-next.jgi.doe.gov/blast-search). Significant (1e^−30^) hits typically corresponded to a known homeologous group (Kantarski *et al.* 2016). Two-LOD intervals for linkage mapping were plotted from the combined analysis only and overlapping intervals that were significant across traits and years within an environment were collapsed into a single QTL for visualization purposes.

### Pedigree relationships between NAM parents

Pedigree records from the UMN Breeding Program were used to identify maternal grandmothers and mothers to the NAM Parents. As these generations were completed with random, uncontrolled mating, the male parents were initially unknown. Using historical GBS sequencing data from UMN Cycle 2 combined with GBS data from this study, we identified male parents using Cervus (Kalinowski *et al.* 2007). The TLI Breeding program pedigrees were used to trace the origin of maternal parents to the initial breeding cycles at TLI (Zhang *et al.* 2016).

## Results

We developed a NAM population of IWG with 10 donor lines and one common parent plant with a total of 1,168 genets. To enable replicated observations, these heterozygous plants (genets) were cloned with two replications planted in two contrasting IWG growing environments, MN and KS. Phenotypic observations for flowering time, including spike emergence percent and anthesis timing were recorded in 2017 and 2018.

### Growing degree days

GDD began accumulating earlier at TLI compared with STP (Supplementary Figure S1). Spike emergence data were collected when a visual inspection of the field suggested that spikes were on average approximately 50% emerged from the boot, which occurred on June 1 (GDD: 1567) and June 7 (1599) at TLI and June 8 (854) and 7 at STP (794) in 2017 and 2018, respectively. Anthesis notes were taken when approximately 50% of the plants were beginning anthesis on June 10 (1785) and June 6 (1574) at TLI and June 26 (1219) and June 21 (1111) at STP in 2017 and 2018, respectively. TLI had a severe drought in 2018, which began towards the end of 2017 and lasted through anthesis in 2018. In combination with high temperatures, this drought resulted in plant stress and highly variable maturity where emergence and anthesis occurred simultaneously in many cases.

### Phenotypic data

**Figure 1:**
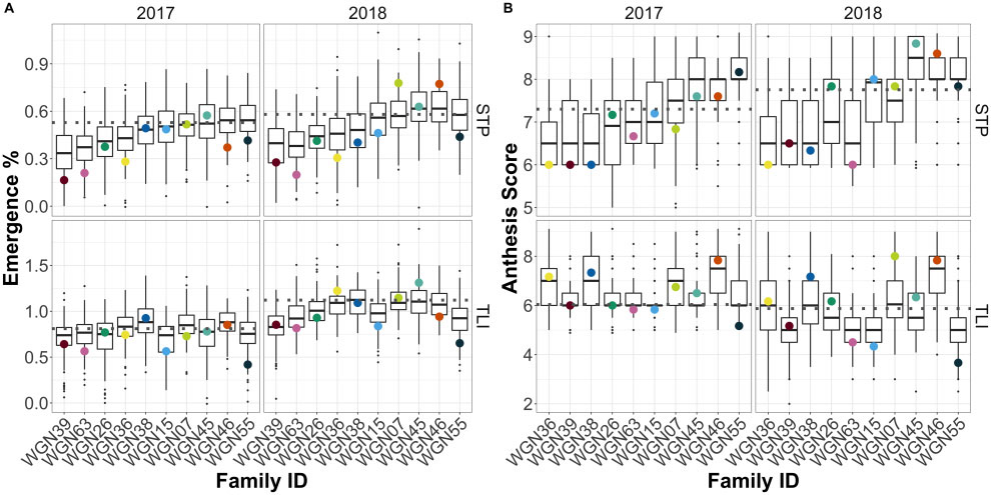
Distribution of progeny emmeans within the 10 IWG NAM families for emergence percent (A) and anthesis score (B) at St. Paul (STP) and the Land Institute (TLI) in 2017 and 2018. Black horizontal lines within boxplots are progeny means. Horizontal gray dotted line indicates common parent mean and colored dots indicate parent means. Families are ordered based on their ranking for STP 2017.

Rankings of family means were mostly consistent across environments and traits (Figure 1, A and B), with families 39, 63, 26, and 36 being the latest, and 55 being the earliest. The common parent (WGN59) was typically earlier to emerge and similar for anthesis timing relative to the other parents. The maximum difference between parental phenotypes averaged 0.40 for spike emergence (40% of spike emerged), and 1.75 growth stages for anthesis (difference between a few anthers showing and ∼75% of anthers showing). The donor parent with the most divergent phenotype varied depending on the environment and trait, and by and large, divergence in parental phenotypes of a cross was not a significant predictor variation among progeny, with the exception of anthesis score in TLI 2017 (*r *=* * 0.84; *P *=* * 0.002; Supplementary Table S2). Furthermore, in every case, the range of progeny phenotypes within families was much greater and exceeded that of the parents, providing evidence for transgressive segregation (Figure 1, A and B). Emergence and anthesis were positively associated in all environments (Supplementary Figure S3). In STP 2017 and 2018, where emergence percent data were recorded around 50% emerged, the trend between the two traits was linear (*P *<* * 0.0001). At TLI in 2017 and 2018, when data were recorded later, around 75%–100% emerged, the relationship was best described by a quadratic fit (*P *<* * 0.0001). In all environments, but especially at TLI, within each category of anthesis stage, spike emergence varied widely, suggesting that early emergence is not always associated with early anthesis. There were no notable significant maternal effects for either trait, with the exception of one instance, where progeny derived from WGN38 showed a significantly lower anthesis score than progeny derived from the common parent in 3 of 4 environments (Supplementary Tables S3 and S4). Progeny derived from self-pollination tended to be significantly later maturing according to both measures (Supplementary Figure S4).

### Marker data and SNP filtering

**Figure 2:**
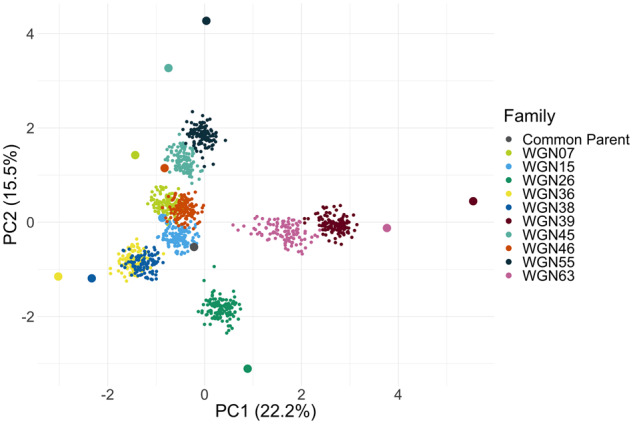
Principal components (PC) analysis of the intermediate wheatgrass nested association mapping population families, where large dots indicate parents, small dots indicate progeny and colors indicate family identity. Axis labels include percent variance explained for the two PCs.

Initial SNP marker count was 444,023 which was reduced to 8,003 after filtering. Two genets were removed because they had greater than 70% missing marker data. The common parent had a high propensity for self-pollination with a rate of 8.3% (range of 1.5%–15.9%, depending on the family), while the donor parents averaged 2.9% (range of 0%–11.4%, depending on the family). A total of 74 individuals were removed from the final analysis because they were identified as progeny derived from self-pollination or unintended outcrosses. Final family size on average was 117 individuals. Imputation accuracy for LinkImpute was 95.3%. Linkage disequilibrium, as defined by *r*^2^ = 0.2, varied across linkage groups with a range of 14.5 cM for LG 20, and 53.5 cM for LG 18 with a median of 21.08 cM (Supplementary Figure S5). The first two principal components of the genotype matrix explained 22.2% and 15.5% of the variation (Figure 2). The distribution of individuals, with the common parent in the center, and progeny distributed approximately mid-way between parents, demonstrated the expected relationship (either half- or full-sibs) between individuals in the population.

### Consensus linkage map creation

**Figure 3:**
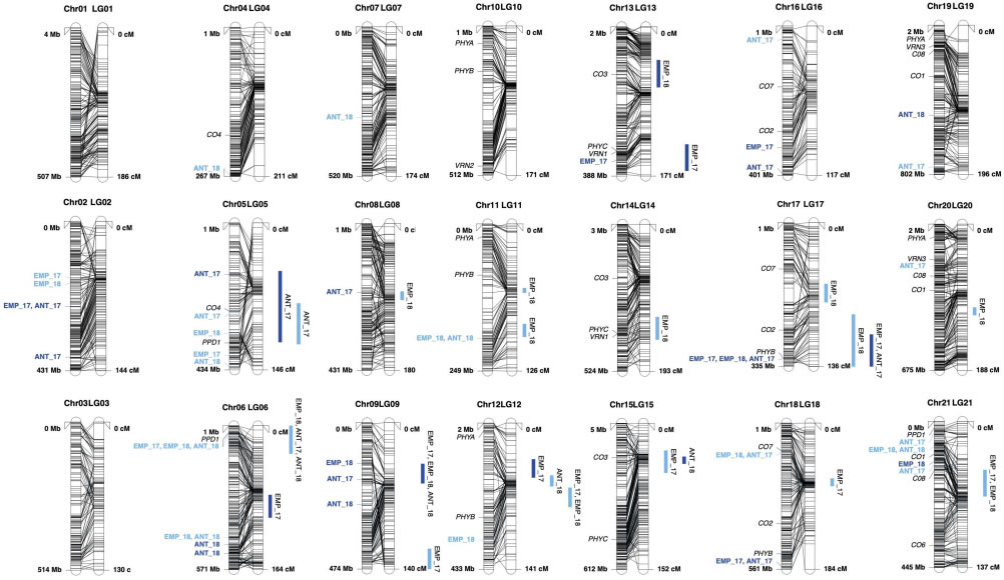
For each chromosome, the physical map (unpublished, access provided by the *Thinopyrum intermedium* Genome Sequencing Consortium) is on the left, and linkage maps developed in the present study are on the right. Markers that were used in both mapping approaches are connected with a line. Physical distances in megabase pairs (mbp) and genetic distances in centimorgans (cM) are normalized to comparable lengths. Included on the physical map (left), are the significant markers from GWAS and possible candidate orthologous genes (black italic). Included on the linkage map (right) are the 2-LOD drop off intervals for the combined analysis across populations (bars indicate interval length), for STP (light blue) and TLI (dark blue) for emergence percent (EMP) and anthesis (ANT) followed by the years (17 and 18 for 2017 and 2018) in which the marker interval was detected.

After filtering for MAF and missing data within families, an average of 3,003 markers per family (average 143 per LG) were used for initial linkage mapping analysis. Across families, 40% (range: 36%–43%) of markers displayed significant segregation distortion at α** **=  0.1, and were excluded from the map making step (Supplementary Figure S6). A greater proportion of the hkxhk type markers (average 71%) exhibited distortion, followed by lmxll (28%), and nnxnp (24%). In the case of LG 18, there was an insufficient number of undistorted lmxll (common parent, WGN59) markers to contribute to the map and thus the map for LG 18 only includes nnxnp markers. The final map length averaged 161 cM per LG with a total length of 3,385 cM (Haldane’s mapping units) and a density of one marker per 0.93 cM (Figure 3). Pearson correlation was used to assess the quality of the map order using markers in common with the consensus genetic map (Kantarski *et al.* 2016) and 17 LGs were above *r *= 0.95, with four having lower correlations (Supplementary Figure S7). Markers that exhibited distortion in one family, but were included in map making in another, were allowed to phase in the creation of the .loc files for use in MapQTL. There were approximately 1,652 markers per family in common between the consensus and the final NAM map with an average of 78 per LG (Supplementary Figure S8). To implement the TWPT, hkxhk markers were excluded which reduced this number to an average of 994 markers per family and 47 markers per LG.

### Genome wide association

Nineteen and twenty-six marker-trait associations were detected for the emergence percent and anthesis score respectively across the four environments using GWAS (Figure 3; Table 2). Seven were significant in two trait environment combinations, and two in three environment trait combinations. Markers generally explained a small percent of the variation for a trait, for a median of 1.7 and 1.9 for emergence and anthesis, respectively. Allele effects corresponded to a median absolute difference of 6.5% spike emerged, or a 0.35 fraction of a growth stage. Most QTL were detected on chromosomes 5, 2, 6, 21, 9, and 16. Most QTL were detected at STP 2018 (15) and fewest were detected at TLI 2018 (7).

**Table 2:** Results from genomewide association mapping results for all environments organized by trait and environment (top row). Instances in which the QTL was not detected in the present environment are indicated by missing values (“—").

					STP 2017			STP 2018			TLI 2017			TLI 2018		
Trait	**SNP** ^a^	**Alleles** ^b^	**MAF** ^c^	**Segregating Families** ^d^	**−log_10_(p)** ^e^	**Effect** ^f^	**PVE** ^g^	−log_10_(p)	Effect	PVE	−log_10_(p)	Effect	PVE	−log_10_(p)	Effect	PVE
Emergence Percent	Chr02_163115345	T/C	0.11	15, 63	3.93	–0.07	3.65	—	—	—	—	—	—	—	—	—
	Chr02_178305818	T/C	0.16	26, 38, 39, 45, 55, 63	—	—	—	3.66	0.05	0.84	—	—	—	—	—	—
	Chr02_245307556^h^	C/T	0.02	38	—	—	—	—	—	—	5.2	0.18	1.44	—	—	—
	Chr05_327412330	A/G	0.05	36, 39	—	—	—	3.87	–0.07	1.44	—	—	—	—	—	—
	Chr05_392431630	C/G	0.05	39, 63	3.68	–0.07	1.63	—	—	—	—	—	—	—	—	—
	Chr06_27073072^h^	T/C	0.44	All	4.27	0.03	2.34	9.53	0.06	3.27	—	—	—	—	—	—
	Chr06_445976596^h^	G/T	0.34	07, 15, 38, 45, 46, 55	—	—	—	7.1	–0.06	1.05	—	—	—	—	—	—
	Chr09_131144770	A/G	0.26	07, 36, 39, 45, 46, 63	—	—	—	—	—	—	—	—	—	4.36	–0.06	2.41
	Chr11_193858632^h^	G/A	0.05	39, 55	—	—	—	3.87	–0.07	0.58	—	—	—	—	—	—
	Chr12_347517636	A/G	0.13	26, 39, 45, 55, 63	—	—	—	3.75	0.05	0.91	—	—	—	—	—	—
	Chr13_347308338	C/T	0.13	15, 38, 39	—	—	—	—	—	—	3.66	–0.08	0.82	—	—	—
	Chr16_326253169	G/C	0.47	All	—	—	—	—	—	—	3.82	–0.05	1.67	—	—	—
	Chr17_317374690^h^	G/C	0.36	All	—	—	—	—	—	—	4.25	–0.05	2	3.81	–0.04	2.01
	Chr18_112460873^h^	G/A	0.05	36, 38	—	—	—	4.79	–0.08	1.7	—	—	—	—	—	—
	Chr18_550223545^h^	G/C	0.16	07, 36, 38, 39, 45, 55	—	—	—	—	—	—	5.17	0.07	1.76	—	—	—
	Chr21_75063069^h^	T/A	0.19	07, 36, 38, 46, 63	—	—	—	3.7	–0.05	2.46	—	—	—	—	—	—
	Chr21_121306193	C/T	0.02	07, 15	—	—	—	—	—	—	—	—	—	4	0.1	0.78
Anthesis Score	Chr02_245307556^h^	C/T	0.02	38	—	—	—	—	—	—	5.47	0.79	2.82	—	—	—
	Chr02_391596222	G/A	0.15	07, 38, 45, 46	—	—	—	—	—	—	3.93	0.3	2.03	—	—	—
	Chr04_266676626	T/C	0.15	26, 39, 45, 46, 55, 63	—	—	—	3.81	0.26	1.53	—	—	—	—	—	—
	Chr05_152472752	T/C	0.12	45, 46, 55	—	—	—	—	—	—	4.48	–0.35	3.15	—	—	—
	Chr05_269372528	C/T	0.02	26	5.96	0.75	2.91	—	—	—	—	—	—	—	—	—
	Chr05_426524999	G/C	0.25	15, 26, 36, 38, 39, 45, 46	—	—	—	4.11	–0.25	1.77	—	—	—	—	—	—
	Chr06_27073072^h^	T/C	0.44	All	—	—	—	10.02	0.32	4.08	—	—	—	—	—	—
	Chr06_445976596^h^	G/T	0.34	07, 15, 38, 45, 46, 55	—	—	—	6.37	–0.32	0.76	—	—	—	—	—	—
	Chr06_470381731	G/T	0.45	All	—	—	—	—	—	—	—	—	—	5.58	0.28	2.07
	Chr06_507506304	C/T	0.40	All	—	—	—	—	—	—	—	—	—	3.65	0.28	0.54
	Chr07_315344031	C/T	0.05	39, 55	—	—	—	4.25	–0.44	0.08	—	—	—	—	—	—
	Chr08_202486528	T/C	0.05	07, 46	—	—	—	—	—	—	4.12	–0.49	0.93	—	—	—
	Chr09_183303350	C/T	0.02	46	—	—	—	—	—	—	4.48	0.65	0.99	—	—	—
	Chr09_265159585	C/T	0.03	07	—	—	—	—	—	—	—	—	—	3.67	0.72	0
	Chr11_193858632^h^	G/A	0.04	39, 55	—	—	—	4.94	–0.46	0.52	—	—	—	—	—	—
	Chr16_34501928	T/C	0.05	26, 55	3.76	0.36	0.4	—	—	—	—	—	—	—	—	—
	Chr16_397627839	G/A	0.39	All	—	—	—	—	—	—	3.89	0.2	2.37	—	—	—
	Chr17_317374690^h^	G/C	0.36	All	—	—	—	—	—	—	5.32	–0.24	1.9	—	—	—
	Chr18_112460873^h^	G/A	0.05	36, 38	4.05	–0.41	1.78	—	—	—	—	—	—	—	—	—
	Chr18_550223545^h^	G/C	0.16	07, 36, 38, 39, 45, 55	—	—	—	—	—	—	4.85	0.27	1.95	—	—	—
	Chr19_484430789	T/C	0.03	07	—	—	—	—	—	—	—	—	—	4.27	0.78	1.85
	Chr19_762292265	C/T	0.07	15, 39, 45	4.78	0.38	1.98	—	—	—	—	—	—	—	—	—
	Chr20_193202832	C/T	0.11	15, 45, 55	3.73	0.35	0.5	—	—	—	—	—	—	—	—	—
	Chr21_33179355	G/A	0.12	26, 36, 38, 55, 63	3.98	–0.28	2.52	—	—	—	—	—	—	—	—	—
	Chr21_75063069^h^	T/A	0.19	07, 36, 38, 46, 63	—	—	—	4.76	–0.34	3.06	—	—	—	—	—	—
	Chr21_131911561	G/C	0.3	07, 36, 38, 39	4.36	0.33	3.8	—	—	—	—	—	—	—	—	—

a SNP, single nucleotide polymorphism, including chromosome number followed by position in base pairs.

b Reference and alternate alleles.

c Minor allele frequency within the entire 10-family NAM population.

d NAM families in which the allele segregates above a frequency of 0.05.

e Level of significance.

f Allele effect in trait units, associated with the alternate allele.

g PVE, percent variance explained by the QTL.

h Indicates a SNP detected in both traits.

### QTL linkage mapping

In the combined analysis, 24 and 8 QTL intervals detected for emergence percent and anthesis score, respectively. Of these, five were identified in more than one trait or environment (Figure 3; Table 3), and the majority (62.5%) were identified as segregating in the donor parent. In the individual family analyses, the most QTL were detected in families 15 (10), 26 (10), and 36 (10). A QTL detected in the combined analysis typically overlapped anywhere between zero and five significant within-family QTL (Table 3). Allele effects and variance explained are only calculated in the within family analyses. The median variance explained for a QTL was 14.8% (range 8.6%–73.3%). The most QTL were found on LG 6 (12 in unique analysis by trait by environment combinations), LG 17 (11), and LG 12 (10).

**Table 3:** Results from linkage mapping analyses combined across families and within families for emergence percent and anthesis, including two-LOD drop off intervals, peak loci, maximum LOD, variance explained and allele effects.

Trait	Location	Year	**LG** ^a^	**Parent** ^b^	Left^c^	Peak^d^	Right^e^	**Peak locus** ^f^	Max LOD	**Analysis** ^g^	*r* ^2^	**μ** ^h^	**α** ^i^	**γ** ^j^
Emergence Percent	STP	2017	6	Donor	0	18.6	31.9	DP_Chr06_27073118	11.3	combined	—	—	—	—
			9	Common	116.8	122.5	140.2	CP_Chr09_130411564	3.93	WGN15	0.10	0.49	–0.05	—
			9	Common	120.8	134	140.2	CP_Chr09_95651880	8.19	combined	—	—	—	—
			12	Common	62.2	75.1	80.8	—	9.6	combined	—	—	—	—
			15	Donor	28.4	36.5	51.5	DP_Chr15_158935699	9.09	combined	—	—	—	—
			15	Donor	37.9	43.8	60.4	DP_Chr15_407790725	5.65	WGN15	0.15	0.49	—	0.06
			18	Donor	58.4	76.8	93.3	DP_Chr18_170459443	3.85	WGN36	0.12	0.42	—	0.05
			18	Donor	69.6	72.7	79.2	DP_Chr18_120939654	9.2	combined	—	—	—	—
			20	Common	67.1	67.1	89.2	CP_Chr20_276537892	3.24	WGN36	0.10	0.41	0.04	—
			21	Donor	58	65.4	67	DP_Chr21_87225445	9.87	combined	—	—	—	—
		2018	4	Common	140.6	155.3	155.3	CP_Chr04_261391029	3.79	WGN38	0.13	0.47	0.05	—
			6	Donor	0	24.9	28.9	—	19.25	combined	—	—	—	—
			6	Donor	0	26.1	33.6	—	5.05	WGN15	0.18	0.54	—	0.07
			6	Donor	0	25.1	30.9	DP_Chr06_445976596	6.65	WGN55	0.21	0.57	—	0.07
			8	Common	61	87.4	130.7	CP_Chr08_191903001	3.93	WGN36	0.13	0.46	–0.06	—
			8	Common	83.4	87.4	93.8	CP_Chr08_191903001	11.86	combined	—	—	—	—
			11	Donor	52.2	56.9	59.3	DP_Chr11_90001198	8.57	WGN45	0.28	0.64	—	0.08
			11	Donor	54.9	56.9	58.9	DP_Chr11_90001198	14.47	combined	—	—	—	—
			11	Common	85.6	87.4	96.6	CP_Chr11_223223131	10.02	combined	—	—	—	—
			12	Common	34.9	36.2	41.2	CP_Chr12_51058733	4.7	WGN26	0.15	0.45	0.04	—
			12	Common	44.7	55.7	73.1	CP_Chr12_147552391	4.06	WGN15	0.12	0.55	–0.06	—
			12	Common	56.3	59.9	61.8	CP_Chr12_305515847	4.99	WGN36	0.15	0.46	0.06	—
			12	Common	63.6	68.8	88.8	CP_Chr12_332128678	3.84	WGN07	0.10	0.57	0.05	—
			12	Common	68.2	69.2	71.7	CP_Chr12_262720774	19.06	combined	—	—	—	—
			14	Donor	121.5	136.6	151.1	DP_Chr14_409417450	8.43	combined	—	—	—	—
			17	Common	57.9	70.1	75.2	CP_Chr17_97190635	12.7	combined	—	—	—	—
			17	Donor	86.7	103.5	135.7	DP_Chr17_315762187	8.82	combined	—	—	—	—
			20	Common	106	114	121.7	CP_Chr20_537718179	3.99	WGN26	0.14	0.44	–0.04	—
			20	Common	107	114	116.9	CP_Chr20_537718179	11.03	combined	—	—	—	—
			21	Donor	45.5	60.6	70.3	DP_Chr21_113736059	8.23	combined	—	—	—	—
			21	Donor	70.3	87.3	92	—	4.86	WGN63	0.19	0.38	—	–0.06
	TLI	2017	6	Donor	74.4	91.5	106.8	DP_Chr06_367267076	4.21	WGN36	0.13	0.81	—	–0.06
			6	Donor	78.9	92.5	104.8	—	11.74	combined	—	—	—	—
			6	Donor	85	114	144.8	DP_Chr06_490834190	3.52	WGN15	0.13	0.69	—	0.07
			9	Donor	39.4	47.1	52	DP_Chr09_59953210	8.26	combined	—	—	—	—
			12	Common	34.9	49.2	52.5	—	11.59	combined	—	—	—	—
			12	Common	61.8	62.4	67.6	CP_Chr12_208165297	5.72	WGN36	0.16	0.82	0.07	—
			13	Donor	134.9	151.5	165.3	DP_Chr13_328372421	9.82	combined	—	—	—	—
			13	Donor	134.9	157.3	166.3	—	6.52	WGN38	0.22	0.87	—	–0.10
			15	Donor	33.2	58.1	86	—	5.19	WGN15	0.16	0.68	—	0.08
			17	Donor	99.5	126.3	135.7	—	3.45	WGN38	0.09	0.88	—	–0.07
			17	Donor	105.5	126.3	135.7	—	10.41	combined	—	—	—	—
			17	Common	106.2	117.4	135.7	CP_Chr17_321627070	4.67	WGN55	0.15	0.74	0.08	—
			17	Common	115.3	125.8	135.7	—	14.8	combined	—	—	—	—
		2018	9	Donor	46.1	52	58.1	—	8.14	combined	—	—	—	—
			13	Common	35.6	59.2	70.4	CP_Chr13_104225517	3.68	WGN26	0.13	1.00	0.06	—
			13	Common	35.6	92.1	150	—	3.84	WGN55	0.24	0.91	0.11	—
			13	Common	38.2	48.6	69.4	—	9.69	combined	—	—	—	—
			14	Donor	25.9	25.9	47.2	DP_Chr14_2538957	3.62	WGN36	0.14	1.09	—	0.06
			21	Donor	52.4	61.4	71.5	DP_Chr21_90120652	3.44	WGN36	0.13	1.08	—	–0.06
Anthesis Score	STP	2017	5	Donor	38.2	101.8	125.8	—	9.8	WGN26	0.73	7.00	—	–0.69
			5	Donor	80.1	109.2	120.8	DP_Chr05_385257903	14.89	combined	—	—	—	—
			6	Donor	0	27.1	39.5	—	5.06	WGN55	0.17	8.10	—	0.27
			15	Donor	37.9	39.3	67.7	DP_Chr15_357630002	4.69	WGN15	0.17	7.08	—	0.33
			21	Donor	33.7	64.8	85.3	—	3.69	WGN07	0.13	7.30	—	0.31
		2018	5	Donor	6.1	96.9	122.8	—	7.1	WGN26	0.66	7.16	—	–0.65
			6	Donor	0	24.9	29.9	—	14.46	combined	—	—	—	—
			6	Donor	0	24.9	33.6	—	5.44	WGN15	0.19	7.48	—	0.41
			6	Donor	0	22	31.9	—	4	WGN55	0.15	8.00	—	0.28
			12	Common	50.5	56.3	60.9	CP_Chr12_203098219	14.05	combined	—	—	—	—
	TLI	2017	2	Common	12.1	65.3	114.8	—	3.31	WGN26	0.16	6.35	0.31	—
			2	Donor	33.2	58.9	65.2	DP_Chr02_274704624	3.45	WGN36	0.13	7.00	—	–0.39
			3	Donor	0	0	20	DP_Chr03_42742623	5.4	WGN46	0.66	7.42	—	–0.91
			5	Donor	7.1	92.9	118.8	—	6.35	WGN38	0.61	7.08	—	–0.91
			5	Donor	48	60.6	118.8	DP_Chr05_153821474	9.44	combined	—	—	—	—
			9	Common	118.8	134	140.2	CP_Chr09_95651880	2.88	WGN15	0.10	6.09	–0.18	—
			12	Donor	103.8	139.8	139.8	—	5.62	WGN46	0.66	7.42	—	0.90
			13	Donor	129.7	129.8	130.1	DP_Chr13_337044446	5.04	WGN38	0.21	7.03	—	–0.50
			15	Donor	93.5	102.6	102.6	DP_Chr15_521971228	3.97	WGN26	0.13	6.31	—	–0.28
			17	Donor	104.5	126.3	135.7	—	4.29	WGN36	0.16	6.94	—	–0.44
			17	Common	107.2	121	135.7	—	4.33	WGN55	0.16	6.48	0.40	—
			17	Common	109.3	133.8	135.7	CP_Chr17_324713190	3.76	WGN26	0.15	6.33	0.30	—
			17	Donor	113.9	135.7	135.7	DP_Chr17_314914761	15.19	combined	—	—	—	—
			17	Common	121	131.8	135.7	—	21.58	combined	—	—	—	—
			21	Donor	63	70.4	83.5	DP_Chr21_203177933	3.71	WGN26	0.12	6.30	—	–0.28
		2018	6	Donor	122.1	144.8	144.8	—	4.28	WGN26	0.17	5.81	—	–0.44
			9	Donor	44.2	49.2	70.7	DP_Chr09_165133955	3.82	WGN07	0.14	6.23	—	–0.42
			9	Donor	44.4	47.1	56.3	DP_Chr09_59953210	9.51	combined	—	—	—	—
			10	Common	25.4	41.4	75.4	—	3.8	WGN55	0.16	5.21	–0.43	—
			15	Donor	34.7	36.7	42.1	DP_Chr15_118317427	10.94	combined	—	—	—	—
			15	Donor	47.7	64.7	74.9	DP_Chr15_428831151	4.01	WGN15	0.14	5.05	—	0.30
			15	Common	122.1	131.9	131.9	CP_Chr15_571603230	3.66	WGN07	0.11	6.28	0.39	—
			19	Donor	100.8	119	125.3	DP_Chr19_555538398	3.95	WGN63	0.15	5.02	—	0.25

a LG, linkage group.

b Map used in linkage mapping.

c–e Genetic positions in centimorgans (cM) of the 2-LOD drop-off interval.

f Peak locus of the QTL interval where“-“indicates that the peak was between two markers.

g Analysis whether the QTL was detected in the combined (including all families) analysis or within the analysis of a specific family. Allele effects are not reported in the combined analysis.

h Overall mean.

i Difference between alleles in the common parent.

j Difference between alleles in the donor parent.

### Pedigree relationships

Using pedigree records and historical genotype data from the breeding program, we determined multiple shared relationships between the NAM parents, which were initially selected based on phenotype alone (Supplementary Figures S9 and S10; Zhang *et al.* 2016). Importantly, we determined that the common parent, WGN59, was derived from a mating of at least half-siblings (male parents are unknown), leading to a minimum inbreeding coefficient for that individual of F ≥ ⅛. Parent WGN26 is a half-sibling to WGN59 (coefficient of coancestry, *f*_WGN26, WGN59 _≥ ⅛). Parents WGN36 and WGN38 were full siblings, and along with WGN15, they share a common grandmother (C3-3471) with WGN59 (*f*_WGN36, WGN59_ = *f*_WGN38, WGN59_ = *f*_WGN15, WGN59_ ≥ 1/16). Parent C3-3471 was the first nonshattering, 90% free-threshing plant derived from The Land Institute’s breeding program and has been used in numerous crosses in the UMN program and in the creation of the consensus genetic map (Kantarski *et al.* 2016). We also identified interrelatedness among six donor parents that are half-sibs, sharing either a mother or father in common, including: WGN39 and WGN63, WGN07 and WGN46, WGN45 and WGN55.

## Discussion

IWG is currently undergoing domestication as a perennial grain crop for human consumption. Selection targets for this crop are numerous and the understanding of the genetic control of important traits remains relatively unknown. NAM as a method for marker-trait dissection has proven useful in other crops but has not been tested before in a species with this mating system, one that is self-incompatible and requires the use of F_1_ progeny. We examined a 10-family F_1_ NAM of IWG developed with phenotypically diverse parents from Cycle 2 of the University of Minnesota breeding program. Considering the importance of variation in flowering time for optimizing yield and performance in new environments, we sought to increase our understanding of the genetic control of flowering time in IWG and determine the utility of this population for genetic mapping.

Two methods were used to detect QTL for flowering time, GWAS and two-way pseudo testcross linkage mapping using both a combined and within population analysis and a genetic map created specifically for the NAM. In most cases, regions with many GWAS QTL coincided with significant linkage mapping intervals that were consistent across multiple trait and environment combinations (Figure 3). Furthermore, these regions, specifically on chromosomes 6, 17, 14, 5, and 21, were in supported by BLAST hits and corresponding gene models to orthologous barley flowering time genes. On chromosome 6, GWAS marker Chr06_27073072, which was detected in in both STP years for emergence percent and for anthesis in STP 2018 is 23.9 kb away from a significant BLAST hit and corresponding gene model for the well-characterized *Ppd-H1* gene in barley that delays flowering time (Turner *et al.* 2005; Figure 3). This QTL region is further supported by the QTL linkage mapping results, which was significant in three analyses in STP. In GWAS, the allele segregated in all ten families, and explained on average 3.23% of the variation (Table 2). In linkage mapping, this region was detected in the combined analysis and in families 15 and 55 where it explained on average 19% of the variation, suggesting that this marker may have family specific effects (Table 3). A TLI-specific QTL on chromosome 17 was detected in GWAS (Chr17_317374690) in three of four TLI analyses and was supported by an overlapping QTL interval (peaking around an average of 127 cM) that was detected in two of four analyses at TLI and one at STP. The GWAS QTL was within 66 mpb and 57 kb from significant BLAST hits for the orthologous genes *Constans 2* and *PHYB*, respectively (Griffiths *et al*. 2003; Szűcs *et al.* 2006). Though only detected using linkage mapping in emergence percent at STP in 2018, a QTL interval on LG 14 aligned closely with a hit for *PHYC* and *VRN1* (Fu *et al.* 2005; Nishida *et al.* 2013). On chromosome 5, we detected, across both approaches and in multiple environments, QTL that aligned near orthologs of *Constans4* and *Ppd-H1* (Griffiths *et al.* 2003; Turner *et al.* 2005), and QTL on chromosome 21 near *Constans1* and *Constans8* (Figure 3; Griffiths *et al.* 2003). These results are the first to provide evidence that the well-described flowering time pathways in barley may also play a role in determining flowering time in IWG. We also identified regions that aligned closely previously described maturity QTL in IWG on chromosomes 12 and 17 (Larson *et al.* 2019). The fact that the NAM population revealed new QTL, previously described QTL, and newly identified QTL that align closely with likely orthologs, suggests that the NAM approach was effective at mapping flowering time in IWG.

It is important to note, however, that there was some discrepancy between the two mapping methods. For example, GWAS QTL on chromosomes 2, 16, and 18 (Figure 3) were not found in linkage mapping, and vice versa for QTL on chromosomes 8, 12, 17, and 20. The IWG reference genome is still in development and thus it is likely that sequences may be out of place, as is evidenced by some discrepancy in marker order from the genetic and physical maps (Figure 3). Furthermore, mapping resolution may have been limited due to few recombination events in the development of the population. The severe segregation distortion and high LD identified in the population may have also limited mapping resolution in the linkage mapping method, which we describe in further detail below.

The results also differed in some cases across environments, which suggests that different pathways or genes influence flowering time in different environments. For example, the QTL on chromosome 6 in the region of *Ppd-H1* were only detected in STP, and those on chromosome 17 were found primarily at TLI. This finding is supported by the vast differences in GDD accumulation across the two sites (Supplementary Figure S1). Furthermore, previous work in IWG has also shown that the presence and location of QTL can vary depending on whether phenotypic data are tested on an individual environment basis or averaged across environments (Larson *et al.* 2019; Mortenson *et al.* 2019).

While the NAM population showed promise for its ability to dissect the genetic control of an important agronomic trait, it also had several challenges. First, the parents were selected initially based on their divergent phenotype, but this alone proved to be an inadequate way to select for genetically divergent parents. The purpose of identifying genetically divergent parents would be to maximize the variation observed in the progeny. However, for the purposes of genetic mapping in an F_1_ outcrossing species it is more important to identify parents that are highly heterozygous so their segregations are effective and can be observed. In the case of this NAM, this was hindered by the fact that the common parent, being a progeny of a cross between a minimum of half-siblings likely resulted in increased identity by descent (IBD) and potentially masked recombination. This became apparent in the map making step when certain families and LGs could not be phased, additionally there was severe segregation distortion which reduced the markers that could be used and limited the number of markers that could be tested within the common parent using the TWPT method. This can also likely be observed in high LD that was identified across chromosomes. Previous work in IWG has determined LD to decay to an *r^2^* = 0.20 at 1–5 cM (Zhang *et al.* 2016; 2017; Bajgain *et al.* 2019a), which is much lower than the present estimate of 21 cM. This LD is also higher than one would expect in an outcrossing species (Flint-Garcia *et al.* 2003). This is due in large part to the population design, where the individuals are all related to one another (as opposed to a traditional, diverse GWAS population where only historical recombination is detectable), the inbred nature of the common parent, and the shared pedigree with several of the donor parents (Supplementary Figure S1). LG 18 had the highest LD, indicating that in either recombination could not be detected due to homozygosity within the parents, or that recombination rates were very low. Recombination rates have shown to be highly variable across chromosomes (*e.g.*, Bauer *et al*. 2013). Thus, it is not surprising that LG 18 would not phase for the common parent in JoinMap.

Difficulty in creating genetic maps in IWG due to inbreeding has also been reported (Kantarski *et al.* 2016), and thus it is suggested that future work in IWG ensures high levels of heterozygosity within the chosen phenotypically diverse parents for linkage mapping, which can be achieved through assessing genotype data of the parents. Kantarski*et al.* (2016) also reported segregation distortion in all component maps in the IWG consensus genetic map with presence in variable LGs. One of their maps, C3-3471 x S, was derived from self-fertilization of one individual and exhibited distortion across 11 of 21 groups. Interestingly, another population, M26 x M35, which was derived from a mating of half-siblings also exhibited high rates of distortion in groups 1, 3, 4, 6, 7, 8, 10, 13, 14, and 20 (Kantarski *et al.* 2016), but was effective in terms of its mapping potential both using the CP and TWPT approaches (Larson *et al.* 2019). The populations used in the present population represent a later breeding cycle and thus there have since been additional opportunities for inbreeding and shared pedigree. Kantarski *et al.* (2016) also reported bias in the segregated markers towards the hkxhk types, which was also found in this case (Supplementary Figure S8) and suggests the presence of lethal or deleterious alleles.

Two methods of measuring reproductive development, spike emergence and anthesis, were assessed in two distinct environments over 2 years. The methods exhibited a positive association with one another (Supplementary Figure S3). Recording anthesis can be challenging in IWG since anthers are on display typically in the evening (∼15:00 hours) and can quickly dehisce with inclement weather such as wind or rain. Once anthers are gone, it becomes very difficult to accurately and efficiently discern between different stages of anthesis. Emergence percent is more time consuming as it involves measuring at least one spike in the field and sometimes more in cases with high variability, as well as spike length after harvest. If spike length is already routinely collected, spike emergence may be an easier method for assessing flowering time, but caution should be taken to measure it around 50% emergence so that a linear relationship exists between the two events. We previously reported that the two measures of flowering time appear to have a slightly different influence on grain yield in spaced plants (Altendorf *et al.* 2020), but no QTL regions detected in the present study appeared to be completely unique to one trait or the other.

In this study, we developed an IWG NAM and investigated reproductive development of IWG. This resulted in the identification many QTL that are located near known orthologous gene models or other previously identified regions. We expect that the QTL detected in this study can be used as fixed effects in genomic selection to alter flowering time in IWG to maximize adaptation to specific environments, or to identify allelic combinations that may lead to more uniformly flowering stands as a way to increase successful pollination. This is especially important as low-floret site utilization has been described as a major barrier to seed yield in IWG (Altendorf *et al.* 2020). In the TLI breeding program, early flowering has been selected as a way of reducing heat and drought stress during seed fill and has provided a yield advantage over later maturing genets. In addition, manipulation of flowering time may be useful in reducing disease pressure such as ergot (*Claviceps purpurea*) which develops in the unfertilized ovaries of cereal crops. In addition to better understanding IWG growth and development, the methods developed in this study should be applicable to a broad range of outcrossing species, where the NAM population design has not been used previously, as well as species that are undergoing domestication for new crop development.

## Data Availability

Supplementary materials and the phenotypic data are available at figshare: https://doi.org/10.25387/g3.13010273. All code and supporting files necessary to reproduce the analyses are available at corresponding author’s GitHub page: https://github.com/kraltendorf under the project name “IWG_NAM_Introduction”. At the time of publication, the parental clones were maintained at the St. Paul Experiment Station in St. Paul, MN, and are available upon request.

## Notes

Mention of trade names or commercial products in this publication is solely for the purpose of providing specific information and does not imply recommendation or endorsement by the U.S. Department of Agriculture. USDA is an equal opportunity provider and employer.
